# The Effect of Hot Working on the Mechanical Properties of High Strength Biomedical Ti-Nb-Ta-Zr-O Alloy

**DOI:** 10.3390/ma12244233

**Published:** 2019-12-17

**Authors:** Dalibor Preisler, Miloš Janeček, Petr Harcuba, Jan Džugan, Kristýna Halmešová, Jaroslav Málek, Anna Veverková, Josef Stráský

**Affiliations:** 1 Department of Physics of Materials, Charles University, 121 16 Prague, Czech Republic; janecek@met.mff.cuni.cz (M.J.); harcuba.p@gmail.com (P.H.); annaterynkova@gmail.com (A.V.); 2 COMTES FHT, 334 41 Dobřany, Czech Republic; jdzugan@comtesfht.cz (J.D.); khalmesova@comtesfht.cz (K.H.); 3 Faculty of Mechanical Engineering, Czech Technical University in Prague, 121 35 Prague, Czech Republic; jardamalek@seznam.cz

**Keywords:** titanium alloys, hot working, biomedicine, tensile testing, fatigue testing

## Abstract

Beta titanium alloy Ti-35Nb-6Ta-7Zr-0.7O (wt%) was developed as a material intended for the manufacturing of a stem of a hip joint replacement. This alloy contains only biocompatible elements and possesses a very high yield strength already in the cast condition (900 MPa). However, the porosity, large grain size and chemical inhomogeneity reduce the fatigue performance below the limits required for utilization in the desired application. Two methods of hot working, die forging and hot rolling, were used for processing of this alloy. Microstructural evolution, tensile properties and fatigue performance of the hot worked material were investigated and compared to the cast material. Microstructural observations revealed that porosity is removed in all hot-worked conditions and the grain size is significantly reduced when the area reduction exceeds 70%. Static tensile properties were improved by both processing methods and ultimate tensile strength (UTS) of 1200 MPa was achieved. Fatigue results were more reproducible in the hot rolled material due to better microstructural homogeneity, but forging leads to an improved fatigue performance. Fatigue limit of 400 MPa was achieved in the die-forged condition after 70% of area reduction and in the hot rolled condition after 86% of area reduction.

## 1. Introduction

Designing a new implant material for total hip replacement with improved properties provides a major market opportunity in the field of medical implants, and, at the same time, a major challenge for material scientists [1]. Several requirements must be fulfilled simultaneously; in particular, the high strength in static loading, acceptable fatigue limit in high cycle fatigue test, positive biological response and sufficient size of initial material for product manufacturing. Beta titanium alloys constitute the most promising and also the most explored material class in this regard [2,3]. Despite years of intensive research, Accolade™ hip implant made from TMZF (Ti-12Mo-6Zr-2Fe) alloy by Stryker Co represents the only commercial β-Ti based hip implant [4,5].

Excellent biocompatibility of titanium was proven by many authors both in vitro and in vivo [6,7,8]. In the last two decades, specialized biocompatible β-Ti alloys have been developed. Among utilizable alloying elements, Nb, Ta, Zr and also Mo are regarded as the most biocompatible, whereas V, Cr and Co are inappropriate [9].

The modulus of elasticity of Ti and common Ti alloys is around 100 GPa, which is half of the elastic modulus of steels, but still much higher than the elastic modulus of the cortical bone (10–30 GPa) [10]. The development of biocompatible β-Ti alloys with reduced modulus of elasticity focused mainly on the Ti-Nb-Ta-Zr alloying system due to the excellent biocompatibility of these alloying elements [7]. Ti-29Nb-13Ta-4.6Zr [11] (Japan alloy, TNTZ) and Ti-35Nb-7Zr-6Ta (US alloy, TNZT) [12] are the two most studied alloys of this type. The latter alloy was developed in USA and patented in 1999 [13]. The alloy was designed empirically by testing many different chemical compositions aiming to achieve the minimum elastic modulus [14]. The elastic modulus of this alloy is below 60 GPa, because α, α’’ and ω phases are avoided in the β solution treated condition. Excellent biocompatibility of the alloy was recently confirmed in [15]. Low strength is the main disadvantage of this class of alloys [16].

Interstitial strengthening by oxygen is one of the major strengthening mechanisms in commercially pure Ti [17]. Interstitial strengthening is efficient also in bcc metals (β phase) [18]. The immense effect of oxygen content on the strength of TNZT alloy was first mentioned in [19]. Moreover, the ductility of this alloy is preserved even for oxygen content of 0.7 wt%, until the alloy contains only β phase. In contrast, in alloys containing α phase (either α + β alloys or metastable β alloys aged to α + β condition), the maximum allowed oxygen content is limited typically to 0.2 wt% to avoid embrittlement [20]. Interstitial strengthening by oxygen has been thoroughly studied in our previous work on as-cast Ti-35Nb-7Zr-6Ta based alloys with various oxygen contents [21].

As-cast Ti-35.3Nb-7.3Zr-5.7Ta-0.7O (TNZTO) alloy contains very large grains with the size ranging from 0.5 to 3 mm. Despite that, the alloy shows promising properties under static loading [22]. On the other hand, fatigue performance of the as-cast material is rather poor, as shown below. 

Investigation of hot working of the alloy is of critical importance not only for improvement of fatigue performance, but also because hot working (namely, die forging) is currently used for implant semi-product manufacturing. Determination of forging conditions is, therefore, of clear practical importance. 

Hot working of β-Ti alloys is done either below or above the beta-transus temperature [23]. The studied TNZTO alloy was deformed in the pure β phase condition. Restoration mechanism in the beta phase field is dominated by dynamic recovery; however, continuous recrystallization in the vicinity of grain boundaries may occur at higher strains [23]. Recovery is a dominant process due to the high stacking fault energy of β-Ti alloys [24].

Hot forging is commonly used for thermomechanical processing of biomedical β-Ti alloys. Ti-36Nb-2Ta-3Zr-0.35O alloy prepared by sintering was successfully hot forged at 800–1150 °C, which caused significant improvement of both the strength and ductility [25]. The authors also claimed that hot forging was comparatively easy due to fine grained structure of the material after sintering. Forging from the diameter of 40 mm to 10 mm at 950 °C was possible in Ti-30Nb-10Ta-5Zr [26]. Ti–xNb–3Zr–2Ta alloy and Ti-35Nb-(10-x)Ta-xZr alloys were successfully forged at 910 °C with 50% reduction and at 900–1000 °C with the reduction of 40%, respectively [27,28]. 

There are also studies concerning the hot working of β alloys intended for aerospace applications. Dynamic recovery is found as a major restoration mechanism in Ti-5553 (Ti-5Al-5V-5Mo-3Cr) alloy during hot deformation [29]. Most studies, however, discuss deformation of Ti-5553 alloy in the α + β field [30,31,32]. In the β field, dynamic recrystallization is preferred to dynamic recovery when high forming temperatures are used [30].

The most important difference between above mentioned alloys and the studied TNZTO alloy is that the TNZTO alloy is much stronger at elevated temperatures, which can be attributed to the strengthening by oxygen. For instance, at the temperature of 800 °C and the strain rate of 0.01 s^−1^, the Ti-5553 alloy deforms already at the stress of approximately 80 MPa [29], while stress of 250 MPa is required for the deformation of TNZTO alloy at these conditions [33]. 

This study aims to develop a manufacturing procedure of the cast material in pure β-phase to achieve a condition suitable for implant production. A sufficiently high temperature of deformation has to be used not only to prevent α phase formation but, more importantly, to reduce the forming forces.

## 2. Materials and Methods 

### 2.1. Alloy Casting

The alloy with nominal composition Ti-35.3Nb-6Ta-7.3Zr-0.7O (wt%) was produced on demand at the company Retech Systems LLC (Ukiah, USA) in the form of a rod with the diameter of 53 mm in two steps. In the first stage, a sponge of Ti and Zr, pieces of Nb and Ta and powder of TiO_2_, were plasma arc melted into compacts with 100 mm diameters, 75 mm lengths and weights of approximately 3 kg. The melting took place under a protective atmosphere of pure He to prevent excessive contamination by oxygen and nitrogen. In the second stage, the compacts were remelted by the sequential pour melting process into a mold with the inner diameter of 55 mm, again in the pure He atmosphere. Each compact was consecutively inserted into melting hearth and poured into mold. The material remains molten at the top but solid at the bottom. The bottom part of the mold can be opened, and with each melted compact added to mold, the ingot is pulled down by about 75 mm. Thus, the process is quasi-continuous and two ingots with the length over 1 m each were produced.

The elemental composition was checked at the top and at the bottom of each produced ingot by the supplier. The content of metallic elements was determined by inductively coupled plasma-atomic emission spectroscopy (ICP-AES) and the contents of non-metallic elements were measured by combustion-infrared absorption (CIA). Results in Table 1 show that the desired nominal composition was achieved. The content of oxygen was accurate and the contents of contaminants (most importantly nitrogen) were negligible.

Altogether, 5 different conditions were produced by two different hot working processes. Two rods with the diameter of 35 mm (area reduction—AR 40%) and 25 mm (AR 70%) were prepared by die forging. Three rods with the diameter of 33 mm (AR 64%), 25 mm (AR 77%) and 20 mm (AR 86%) were manufactured by hot rolling.

### 2.2. Die Forging

Two die-forged conditions were produced at ALPER Co., Prostějov, Czech Republic. In the first stage, pieces of initial ingot were machined to 45 mm and heated to 1220 °C in an electrical laboratory furnace in air for approximately 25 min. Heated pieces were forged to the diameter of 35 mm (AR 40%) by hydraulic press using a closed die of the cylindrical shape (diameter 35 mm) preheated to 300 °C. About 10 forging steps were used during the die-forging, while rotating the forged piece after each step to achieve round cross-section. The whole process took approximately 1 min. One of the forged rods was water quenched (WQ) and the other was put again into the furnace.

This piece was reheated to 1220 °C (within approximately 15 min) and forged to the diameter of 25 mm (AR 70%) using a closed cylindrical die with the diameter of 25 mm. During the forging of the rod with smaller diameter, the temperature of the sides of the forged rod dropped below approximately 800 °C and this piece was reheated back to 1220 °C (5 min) before the final forging to the 25 mm and water quenched. In total, 20 forging steps were used to achieve the piece with the diameter of 25 mm and this piece was reheated three times to reduce diameter from 45 mm to final 25 mm.

The rod forged to 35 mm (AR 40%) was round with the desired diameter, and surface cracks were less than 0.5 mm deep. Material outflow from the die occurred during hot forging to 25 mm. The outflow was machined off. A few surface cracks up to 2 mm in depth were formed.

**Figure 1:**
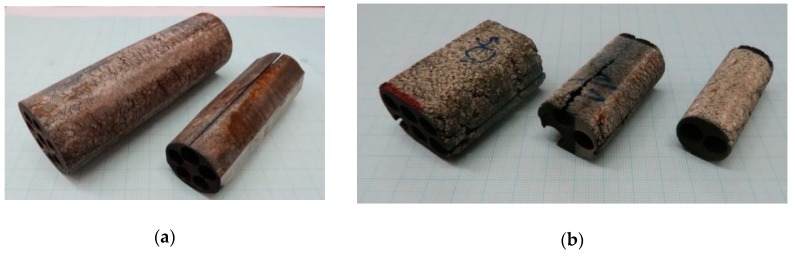
Photographs of (**a**) forged rods and (**b**) rolled rods. The samples for tensile and fatigue testing were already machined from the processed conditions parallel to the axial direction. SEM samples were prepared from cross-section of rods.

Photographs of pieces of both forged rods are shown in Figure 1a.

### 2.3. Hot Rolling

Two pieces of the second manufactured cast ingot (53 mm in diameter) were hot rolled at Technical University Ostrava, Czech Republic. 3 rods with oval cross-sections with larger axes of 33 mm, 25 mm and 20 mm, and smaller axes of 25 mm, 22 mm and 18 mm, respectively, were manufactured. Area reductions were calculated according to actually-achieved cross-sections: 64%, 77% and 86%, respectively. The rolling was performed in a machine with grooves for rolling of round rods along their central axes. The pieces were heated to 1200 °C before the process. Several passes through rolling grooves were used without reheating to achieve the 33 mm diameter and the rods were rotated by 90° after each pass to preserve round or oval shape. One of the rolled rods was water quenched (AR 64%) while the other rod was reheated back to 1200 °C and rolled to the diameter of 25 mm (AR 77%), again in several passes while rotating by 90° after each pass. After rolling to 25 mm, the rod was water quenched and cut into two pieces. One of the pieces was again reheated back to 1200 °C and rolled to the diameter of 20 mm (AR 86%), and finally, water quenched.

Due to the necessity of reheating, the whole process took about 3 h and the rods were heated and processed in air.

The produced rods had a white surface, indicating the presence of titanium/niobium oxide, and large surface cracks up to 3 mm in depth. The shape of the rod rolled with AR 86% was almost round while the other two rolled rods had more oval shapes. The photograph of pieces of produced rods is shown in Figure 1b.

### 2.4. Experimental Techniques

Samples for microstructural observations by scanning electron microscopy (SEM), including electron back-scatter diffraction (EBSD) and energy dispersive X-ray spectroscopy (EDX), and for microhardness measurements, were cut from the forged/rolled rods perpendicular to the axial direction of produced rods. The samples were ground with SiC papers up to grit 2400. Grinding was followed by three-step vibratory polishing employing Alumina 0.3 μm suspension for 6–9 h, Alumina 0.05 μm suspension for 6–9 h and Colloidal Silica for 3–4 h. Field emission SEM FEI Quanta 200FX (FEI, now under Thermo Fisher Scientific, Eindhoven, Netherlands) was used for back-scattered electrons (BSE) observations operating at 10 kV and for EBSD and EDX measurement (both by EDAX, Tilburg, Netherlands) at 20 kV.

Microhardness was measured using Vickers method with the load of 0.5 kgf and dwell time of 10 s.

**Figure 2:**
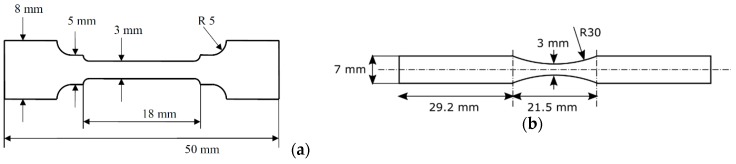
Schematic drawings of (**a**) tensile sample and (**b**) fatigue sample.

Round samples for tensile and fatigue testing were prepared parallel to the axial direction of the forged/rolled rods and at sufficient distance from the surface to avoid the influence of surface cracks or oxidation. Tensile tests of the samples with the gauge length of 15 mm and the diameter of 3 mm were conducted at strain rate of 10^−4^ s^−1^ and 3–4 samples were tested for each condition. Samples for fatigue testing had a typical hour-glass shape with the diameter of 3 mm and the radius of 30 mm. Measurements were done in tension-compression with R = −1 at 50 Hz. Schematic drawings of the tensile and fatigue samples are shown in Figure 2.

## 3. Results and Discussion

### 3.1. Microstructure—Cast Ingot

**Figure 3:**
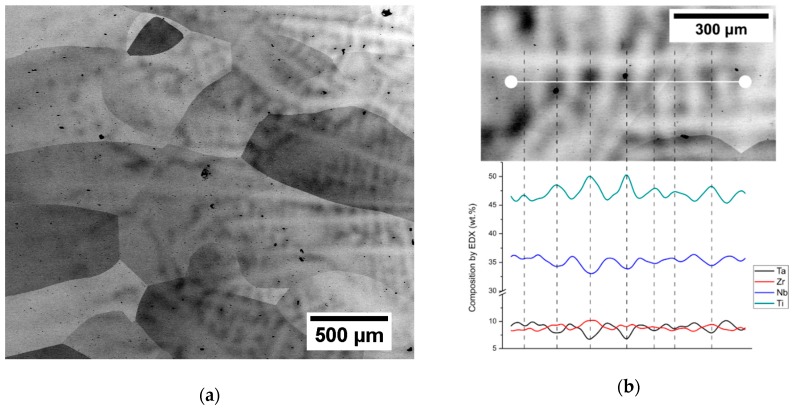
(**a**) SEM micrograph of cast ingot. Left part is closer to the edge; right part is closer to the center of the ingot. More pores and chemical inhomogeneities are seen closer to the center. (**b**) Line scan of composition by EDX along a dendrite.

Figure 3a shows a SEM micrograph of the cast ingot. Very large grains with sizes over 1 mm are present in the material. Porosity appears as black spots with the size of 5–20 µm. In the right half of the image in Figure 3a (i.e., closer to the center of the cast ingot), there are dendritic chemical inhomogeneities, as proven by EDX line scan in Figure 3b. Darker areas are Ti and Zr enriched while lighter areas are Nb and Ta enriched. The compositional fluctuations around mean values are approximately 3%. The chemical inhomogeneity and porosity were located mainly in the central part of the cast ingot.

The alpha phase was not found either in the interior of grains or at the grain boundaries, indicating that the cooling of ingot during production was sufficient to prevent alpha phase precipitation and that the material contains only beta phase. No beta solution treatment was carried out after casting.

### 3.2. Microstructure—Die-Forging

**Figure 4:**
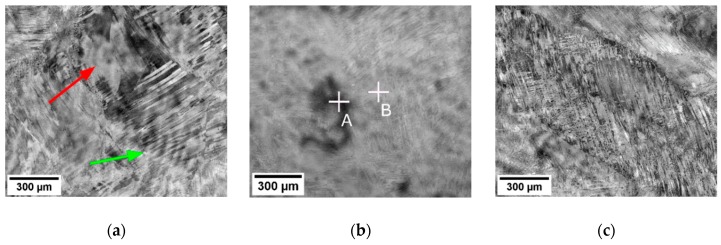
Micrographs of the rod forged with AR 40% showing distinct features: (**a**) non-deformed area (denoted by red arrow) and dendritic inhomogeneity (denoted by green arrow); (**b**) local chemical inhomogeneity; (**c**) grain deformed by slip in parallel directions. Dendritic inhomogeneities can be observed in all micrographs.

Figure 4 shows the microstructure of the rod forged with the AR 40% in three different areas located in the central part of the rod. Note that grains retain the same size as in the cast ingot. On the other hand, they are heavily deformed, as manifested by parallel deformation bands. However, the deformation is not homogeneous and non-deformed regions can also be found (Figure 4a, marked by red arrow). These structures are formed by dislocation slip, as further inspected by EBSD below. Note also that porosity from casting is removed by forging. On the other hand, dendritic chemical inhomogeneities can be still observed, especially in the lower part of Figure 4a (marked by green arrow). Apart from dendritic inhomogeneities, there is a larger chemical inhomogeneity appearing as a darker area in Figure 4b; i.e., Nb and Ta depleted and Ti enriched. The results of EDX analysis of points A and B are shown in Table 2.

**Figure 5:**
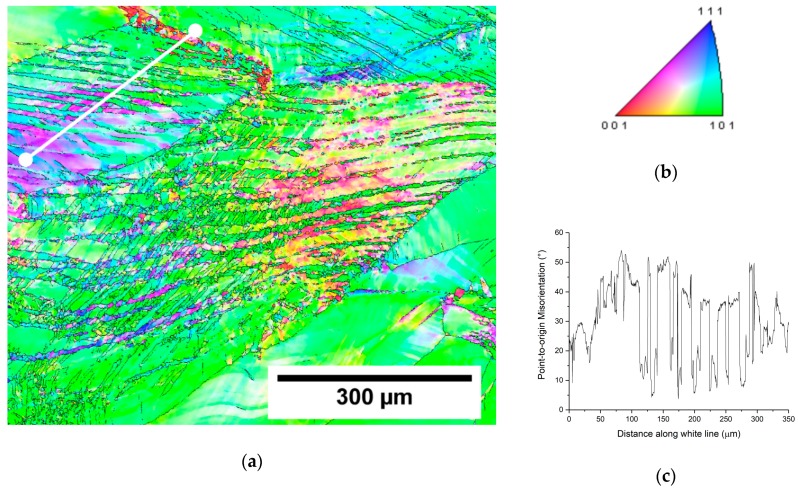
EBSD measurement of the forged rod with AR 40%. (**a**) inverse pole figure (IPF) map, (**b**) orientation triangle (BCC structure) and (**c**) point-to-origin misorientation profile along white line drawn in (**a**).

In order to examine the origin of parallel bands in Figure 4c in detail, the EBSD inverse pole figure (IPF) map of the central part of forged rod with the AR 40% is shown in Figure 5a, along with orientation triangle (Figure 5b) and misorientation profile (Figure 5c) along a line drawn in white in the IPF map. The misorientation profile shows that the misorientations between two grains never exceeds 40°. The misorientation angles of twins occurring in β Ti are 60° in case of {112} <111> twinning and 50.57° for {332} <113> twinning [34]. Therefore, the parallel bands that are intersecting each other are slip bands formed by dislocations. Apart from dislocation bands, significant lattice rotation within the original large grains was observed.

Dynamic (continuous [23]) recrystallization occurred only in the areas with the highest deformation—mainly along grain boundaries of large grains after casting and also along some of the newly formed high angle boundaries.

The microstructure of the rod forged with AR 70% was studied at two different cross-section samples. One was taken from the middle part of the rod, while the other one from the end of the rod (side). While in the forged rod with AR 40%, the orientation of SEM samples was not established, as this rod was round, in the rod forged to AR 70% the direction of last forging step was up/down. The microstructure is completely different from the rod with the AR 40%.

**Figure 6:**
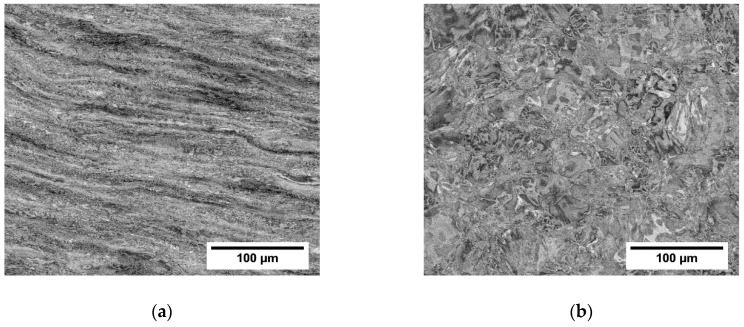
Microstructure of the forged rod (AR 70%) cross sections: (**a**) middle part; (**b**) side of the rod.

In Figure 6a,b the grain structure of the middle and the side of the forged rod with AR 70% are compared (both cross-sections studied showed similar microstructures in their centers and closer to their surfaces, but the middles and sides of the forged rods differ significantly). The microstructure is heavily deformed, as indicated by the channeling contrast of BSE. Due to the high deformation, it is difficult to recognize the individual grains in Figure 6a or Figure 6b. The layered structure observed in the middle part of the rod (Figure 6a) is caused mainly by elongation of grains. The observed contrast is, however, also caused by elongated areas of chemical inhomogeneities (varying Nb content).

**Figure 7:**
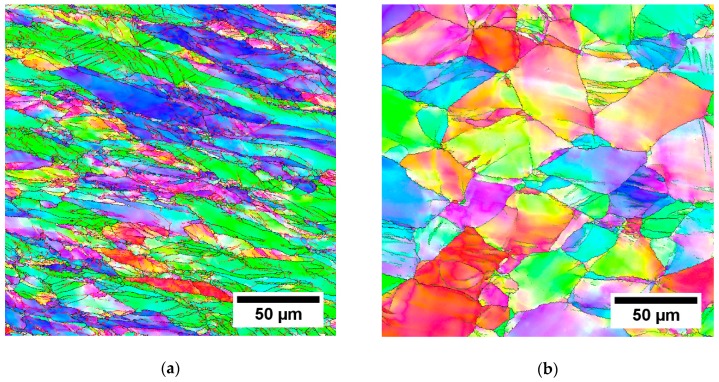
IPF maps of 25 mm forged rod (AR 70%). (**a**) Middle part; (**b**) side of the rod.

EBSD measurements for the rod with the AR 70% are shown in Figure 7 as IPF maps. The difference between the middle and the side of the rod is clearly manifested. The microstructure in the area close to the side of the rod contains equiaxed grains with the size below 50 μm. These grains contain only few deformation bands which indicates that they recrystallized from the deformed structure during the three-step heating and deformation (rods forged with the AR 70% were heated three times before reaching the final diameter, as described in Section 2.2). The grains are, however, slightly deformed, which suggests that the recrystallization itself did not occur in the final deformation step.

In the cross-section from the middle of the rod, the grains are elongated and contain high fraction of low-angle GBs.

### 3.3. Microstructure—Hot Rolling

**Figure 8:**
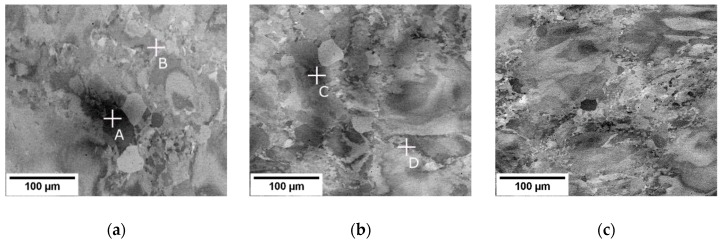
Microstructures of centers of cross-sections in hot rolled rods. Rods with (**a**) AR 64%, (**b**) AR 77% and (**c**) AR 86%.

BSE images of the three hot rolled rods from the centers of the cross-sections are shown in Figure 8. Similarly to the forged rods, no porosity was found in the hot rolled rods. The refinement of microstructure increases with the increasing area reduction, as can be observed mainly by comparing Figure 8a,c. The dark shades in Figure 8a,b are chemical inhomogeneities from casting that were distorted by the rolling procedure, as proven by EDX point analysis of points A, B, C and D, as summarized in the Table 3. Large deformed grains and new small grains can be distinguished in Figure 8, EBSD measurements were performed for a detailed inspection.

**Figure 9:**
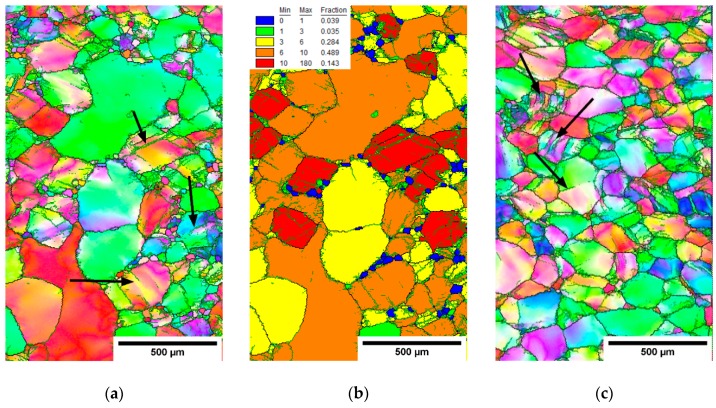
Microstructure of the cross-section of the hot-rolled rod with AR 64%. (**a**) IPF map-center, (**b**) grain orientation spread (GOS) map-center and (**c**) IPF map-edge.

**Figure 10:**
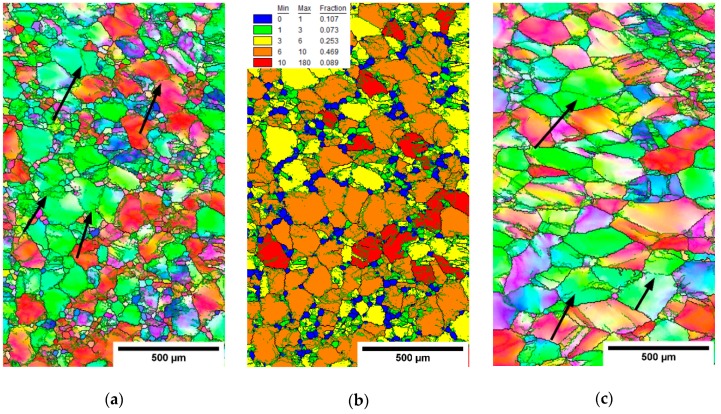
Microstructure of the cross-section of the hot-rolled rod with AR 77%. (**a**) IPF map-center, (**b**) GOS map-center and (**c**) IPF map-edge.

**Figure 11:**
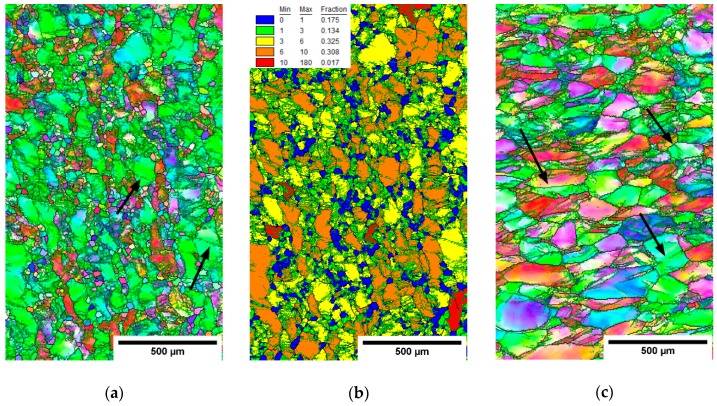
Microstructure of the cross-section of the hot-rolled rod with AR 86%. (**a**) IPF map-center, (**b**) GOS map-center and (**c**) IPF map-edge.

EBSD measurements were performed on the rolled conditions in the cross-section center and edge. Figure 9, Figure 10 and Figure 11 show IPF maps of conditions rolled to AR 64%, 77% and 86%, respectively. In each figure shows (**a**) the IPF map of the center of cross-section, (**b**) the grain orientation spread (GOS) map of the center and (**c**) the IPF map near the edge of cross-section.

The GOS was computed for each grain as a mean deviation of crystalline orientations in the grain from the average grain orientation [35]. In other words, high GOS means that the grain is significantly deformed, and vice-versa: low GOS represents grains with a low deformation—for instance, recrystallized (RX) grains. GOS is normalized to the grain size; i.e., low GOS values are not caused by the small size of grains. The threshold between the GOS values of RX and non-RX fractions was determined on the basis of the histogram of GOS values (not-shown) as 1°, which is a common choice for β-Ti alloys [36]. GOS maps are only shown for centers of each rod as the edges did not contain recrystallized grains with low GOS values.

It is clear from the EBSD images that with the increasing area reduction, the fraction of recrystallized (RX) grains (size of 20–30 µm) increased. New small grains were formed in the areas with higher deformation; namely, at the grain boundaries. This resembles a so-called necklace continuous recrystallization reported in [23]. The recrystallized grains evolve during multiple heating and deformation. Note that there is the lowest RX fraction in the condition with AR of only 64%, which was not reheated. However, the deformed grains also have a smaller size in the rods with higher AR. This can be associated with continuous dynamic recrystallization, which is supported by several incomplete low-angle grain boundaries (marked by black arrows in IPF images), that separate sub-grains.

Quantitative analysis is provided in Table 4, comparing each condition in terms of the grain sizes in both the RX fraction (GOS < 1°) and non-RX fraction (GOS > 1°). In the centers of cross-sections, both the grains from the RX and non-RX fraction are significantly smaller for higher area reductions, while on the cross-sectional edges, the grain sizes are comparable in AR 64% and AR 77% and smaller only in AR 86% condition.

The die-forged rods contained a limited fraction (<1%) of grains with GOS < 1°. The average grain sizes are shown in Table 4 with errors estimated as the distribution widths. Hot-rolled rods showed larger grain size in comparison with die-forged rod with AR 70%. The rod die-forged to 40% was not significantly refined. The dominating restoration mechanism during hot-rolling is recovery. The grains are deformed and their size decreases with larger area reduction. Necklace recrystallization was observed in the centers of rods cross-section. Differences between center and edge are mainly due to temperature, strain and strain rate gradients according to calculations of a similar rolling process [37].

In contrast, deformation bands were observed in the forged rod with AR 40% and only limited recrystallization was present at the grain boundaries. Meanwhile, in a rod forged with AR 70%, fully recrystallized microstructure was present, presumably via continuous recrystallization [23,29]. The grains were equiaxed on side of the rod, while elongated and more refined grains were in the middle of the rod. In comparison with hot-rolled rod with AR 86%, the grain size in rod die-forged to 70% was significantly lower, both on the side and in the middle part. The differences observed between both methods of forming are mainly due to much shorter exposition to the high temperature and much higher strain rate during the forging procedure.

### 3.4. Microhardness Measurements

The microhardness was measured in the as-cast condition and in the forged and rolled rods. The cast ingot exhibited the microhardness of 326 ± 6 HV, while all other conditions showed the microhardness of 344 ± 10 HV. This small though significant difference is clearly caused by thermomechanical processing. On the other hand, the mutual differences in microhardness between hot-worked conditions are negligible, while microstructure differs considerably. This might be caused by the fact that dominant strengthening mechanism in the alloy studied is the interstitial strengthening by oxygen. Other strengthening mechanisms are of lower importance and cause only limited differences in microhardness.

**Figure 12:**
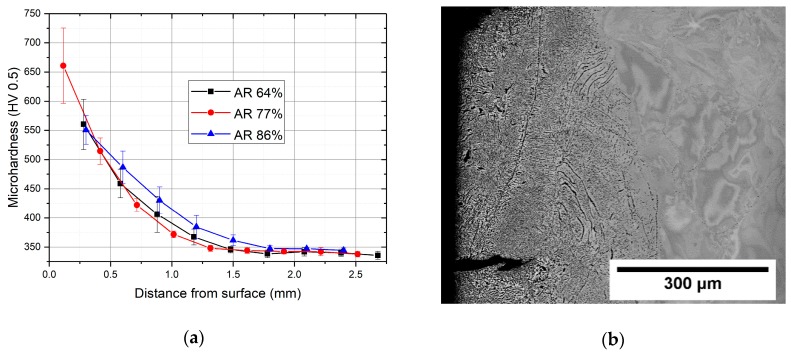
(**a**) Microhardness in the surface region of hot rolled rods; (**b**) SEM image of the α phase precipitated at the surface of hot rolled rod with the AR 86%.

The microhardness of the hot rolled rods near the surface, was much higher than in the bulk, as shown in Figure 12a. Microhardness decreases with increasing distance from the surface for all hot-rolled conditions. In the closest vicinity to the surface, the microhardness exceeds 550 HV, and this value decreases with the increasing distance from surface to the value of 340 HV at 1.7 mm below the surface. The reason for this behavior was undoubtedly the increased oxygen content during exposure to air during the rolling process, which took several hours. Oxygen increases hardness, causes embrittlement in titanium [20] and also stabilizes the α phase. As shown in the Figure 12b, both the α phase precipitation and cracking were observed in the surface region of the condition hot rolled to AR 86%. Note that the α phase precipitates only to a depth of approximately 0.5 mm, while the layer with increased hardness—presumably increased oxygen content—is thicker. The clear practical implication of this behavior is that long-term exposure of the material to air at high temperature should be avoided. For instance, the hot die-forging process descried above was designed to limit the time of exposure to high temperatures.

### 3.5. Tensile Testing

**Figure 13:**
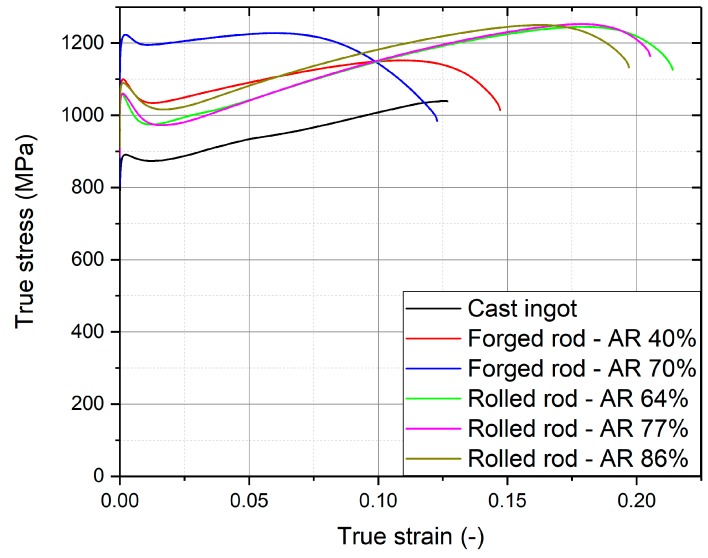
Flow curves of production conditions: cast, forged and rolled rods.

Flow curves from static tensile tests are shown in Figure 13 for all manufactured conditions. Three or four samples were measured for each condition, and representative flow curves (with yield stress, ultimate tensile strength and ductility closest to the average) are shown. Tensile parameters, namely, the yield strength, the ultimate tensile strength and the total plastic elongation, derived from the flow curves, are summarized in Table 5. Extensometer was not used in the measurements and flow curves were determined only from the movement of the cross-head. Elastic parts, were thus subtracted from the true stress–true strain curves to obtain flow curves.

In the cast condition, the upper yield stress was just below 900 MPa; then, the stress slightly decreased (sharp yield point phenomenon), and was followed by a work hardening stage. The flow curve ends with the brittle fracture, apparently without any strain localization (necking) with ultimate tensile strength over 1000 MPa.

Both forged conditions showed higher yield stress and higher ultimate tensile strength, but with considerably larger spread of measured values, as manifested by higher errors in Table 5. The elongation of the forged rod with AR 40% is comparable to the cast condition (approximately 15%) while in the rod with AR 70%, the achieved elongation was only about 10%. At this condition the highest yield strength exceeding 1200 MPa was achieved. In contrast to the cast condition, a significant neck was formed before rupture in the forged samples and was much more pronounced in the rod with AR 70%.

The yield stress of the hot-rolled rods slightly increases with increasing area reduction from 1050 MPa in rod with AR 64% to 1090 MPa in rod with AR 86%. The ultimate tensile strength is approximately 1250 MPa and elongation around 20%. The tensile tests results of the hot-rolled conditions are more reproducible than those from forged conditions. This is consistent with microstructural observations—more homogeneous microstructure in hot-rolled conditions.

The sharp yield points are more pronounced in all deformed conditions in comparison to the cast ingot. In the rolled conditions, the yield points are significantly “sharper” compared to forged conditions. The reason for the occurrence of a sharp yield point was the interaction of dislocations with interstitial oxygen atoms [21,38].

### 3.6. Fatigue Testing

**Figure 14:**
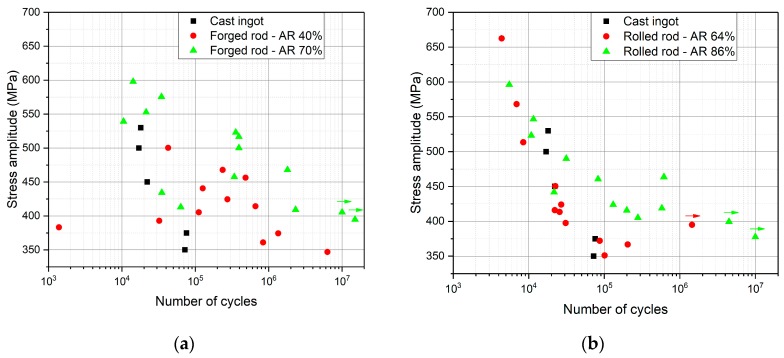
S–N plots of fatigue results: (**a**) cast ingot and die-forged rods; (**b**) cast ingot and hot-rolled rods.

The S–N plots of fatigue tests in tension-compression (R = −1) are shown in two separate graphs for better orientation. Figure 14a shows results from the forged rods compared to the cast condition, while Figure 14b shows results from the hot rolled rods compared to the cast condition. Fatigue properties of the rod rolled to AR 77% were not determined due to insufficient amount of material. In addition, stress amplitude lower than 350 MPa was not used for testing since lower stress amplitudes are not relevant for the foreseen application—manufacturing of the stem of the hip implant.

The cast ingot showed a very poor fatigue resistance, especially when compared with its high strength. The fatigue properties are negatively influenced by very large grain size, by the casting porosity and possibly by the chemical inhomogeneity. Both the die-forging and hot-rolling had removed the porosity and refined the grain structure; therefore, improvement in fatigue properties was expected.

The results of fatigue performance of the two forged rods are not systematic, which can be attributed to the inhomogeneous microstructure. Nevertheless, the rod forged to AR 70% showed generally improved fatigue performance as compared to the rod forged to AR 40%. This clearly correlates with much finer grain structure in the rod forged to AR 70% and its higher tensile strength. The microstructure of the forged rod with AR 70% was, however, not homogeneous, which resulted in the low reproducibility of the fatigue results. The fatigue limit can be estimated as approximately 400 MPa.

The fatigue results of two rolled rods were much more homogeneous. Variations between samples still existed due to differences in the grain size and the fraction of recrystallized grains between the center and surface regions of the hot rolled rods. The hot rolling to AR 64% did not significantly improve fatigue properties. The reduction of the grain size was apparently insufficient. However, the rolling to AR 86% increased the fatigue properties, and in this condition, the fatigue limit could be established as approximately 390 MPa. The reason for this improvement is more homogeneous deformation and refined grain size, together with the absence of the casting porosity.

Rolled conditions showed much more homogeneous grain structures. The forged condition of AR 70% showed, overall, the highest fatigue performance. The most significant differences were seen at stress amplitudes between 450 and 550 MPa, where the lifetime of rolled rods was significantly inferior to the rod forged to AR 70%. This is consistent with the grain size differences between these conditions: the rod forged to AR 70% contains grains of the size of 40 µm on the sides and 15 µm in the middle, while the rod hot-rolled to 86% exhibits grain sizes of the deformed fraction (80% of area) of about 80 µm. However, the effect of microstructure on fatigue properties in the alloy is not fully resolved.

Rolling of a rod to the AR 86% and forging to the AR 70% resulted in the fatigue limits (σ_f_) of 390 MPa and 400 MPa, respectively. However, it constituted only 30% of the UTS values (approximately 1250 MPa for both), while the more common ratio σ_f_/UTS is 0.5 in bcc metals [39]. Studies on fatigue performance of β-Ti alloys in pure β (bcc) conditions are surprisingly scarce. Fatigue limit of 350 MPa was found in Ti-29Nb-13Ta-4.6Zr alloy in the beta solution treated condition [40]. Fatigue limit can be increased to 500 MPa by formation of ω phase or with the use of CeO_2_ particles [41,42]. Low strength alloys Ti-22Nb-6Zr and Ti-24Nb-4Zr-7.9Sn exhibited fatigue limits of 200 MPa and 300 MPa, respectively [43,44]. Fatigue limits in the rotating bending tests of Ti-35.3Nb-7.3Zr-5.7Ta and Ti-35.3Nb-7.3Zr-5.7Ta-0.48O alloys were 280 and 450 MPa, respectively, as reported in [45]. This comprehensive study confirms the $\sigma_{f}$/UTS ratio of 0.5 and demonstrates the effect of the grain size, and, in particular, the average grain misorientation on σ_f_. The initial damage accumulation is caused by coarse planar slip bands which propagate across low angle grain boundaries through several grains [45]. Our microstructural observations showed a high amount of low-angle grain boundaries in the conditions with lower accumulated deformation, and therefore are consistent with this theory. Conditions with the best fatigue performance—as-forged rod with AR 70% and as-rolled rod with AR 86%—are characterized by the smallest grain size and the highest misorientations between grains.

From practical point of view, the diameter of initial material for hip implant manufacturing must be at least 35 mm. Furthermore, the area reduction of approximately 85% is required to achieve satisfactory fatigue performance and oxidized surface layer must be removed after hot working [25]. Therefore, the initial diameter of the cast ingot should be approximately 100 mm.

## 4. Conclusions

Metastable β titanium alloy Ti-35.3Nb-6Ta-7.3Zr-0.7O (wt%) was prepared in several conditions: cast ingot, die-forged rods and hot-rolled rods. The influence of processing route on microstructure evolution, tensile properties and fatigue performance was investigated. The following conclusions can be drawn from this experimental study.

- Casting porosity and very large grains negatively affect mechanical properties, especially fatigue performance.
- Both hot working processes were successful in closing the casting pores.
- Area reduction over 70% for die-forging and over 80% for hot-rolling is required for significant grain size refinement. Apart of achieved strain, recrystallization processes are affected by exact temperature history and distribution of strain rate.
- Tensile properties were improved by both die-forging and hot-rolling.
- Fatigue performance of hot-rolled rods was more homogeneous to that of forged rods due to more homogeneous microstructure, but the rod forged with the AR 70% showed better fatigue performance.

Fatigue limits of the hot-rolled rod with the area reduction of 86% and die-forged rod with the area reduction of 70% were approximately 400 MPa.

## Figures and Tables

**Table 1:** Chemical composition of cast ingots determined by ICP-AES and CIA. Standard errors of measured contents of metallic elements were not determined.

Element (wt%)	Ingot 1-Top	Ingot 1-Bottom	Ingot 2-Top	Ingot 2-Bottom
Titanium	balance	balance	balance	balance
Niobium	35.5	35.6	35.8	35.3
Zirconium	7.36	7.28	7.40	7.36
Tantalum	5.90	6.17	6.01	5.88
Iron	0.049	0.052	0.055	0.050
Oxygen	0.726 ± 0.012	0.702 ± 0.003	0.699 ± 0.002	0.719 ± 0.010
Nitrogen	0.012 ± 0.002	0.012 ± 0.002	0.0108 ± 0.0004	0.012 ± 0.001
Carbon	0.0093 ± 0.0007	0.0101 ± 0.0003	0.0098 ± 0.0009	0.0119 ± 0.0002
Hydrogen	0.0051 ± 0.0001	0.0080 ± 0.0013	0.0061 ± 0.0012	0.0023 ± 0.0001

**Table 2:** Chemical composition (EDX) at positions A and B marked in Figure 3b. Position A is depleted of Nb and Ta.

Position	Ti	Nb	Zr	Ta
A	52.9	31.6	9.4	6.1
B	46.3	36.7	6.8	10.2

**Table 3:** Chemical composition (EDX) at positions A, B, C and D marked in Figure 8a,b. Positions A and C are depleted of Nb and Ta.

Position	Ti	Nb	Zr	Ta
A	51.1	31.3	13.4	4.2
B	45.4	38.4	8.6	7.6
C	49.0	34.8	10.9	5.3
D	50.9	36.8	5.1	7.2

**Table 4:** Comparison of microstructural parameters of each condition produced by hot-rolling and die-forging. The error values shown are the widths of the grain size distributions of each fraction.

**Hot-Rolled Rods**				
**Area Reduction**	**Fraction of Grains with GOS < 1° in Center**	**Grain Size in Fraction with GOS < 1°**	**Grain Size in Fraction with GOS > 1°**	**Grain Size on the Edges**
64%	4%	28 ± 12 μm	270 ± 180 μm	145 ± 65 μm
77%	11%	25 ± 11 μm	114 ± 62 μm	157 ± 66 μm
84%	22%	20 ± 10 μm	78 ± 56 μm	103 ± 64 μm
**Die-Forged Rods**				
**Area Reduction**	**Grain size–Middle Part**		**Grain Size–Side of the Rod**	
40%	0.5 ± 0.3 mm		-	
70%	15 ± 10 µm		38 ± 15 µm	

**Table 5:** Yield strength, ultimate tensile strength and elongation of production conditions.

Condition	Yield Strength (MPa)	Ultimate Tensile Strength (MPa)	Plastic Elongation (%)
Cast ingot	870 ± 25	1039 ± 38	14.8 ± 1.4
Forged rod-AR 40%	1053 ± 71	1129 ± 76	14.1 ± 2.9
Forged rod-AR 70%	1256 ± 68	1264 ± 62	10.4 ± 2.0
Rolled rod-AR 64%	1053 ± 4	1251 ± 4	21.6 ± 0.5
Rolled rod-AR 77%	1078 ± 14	1256 ± 6	20.9 ± 1.2
Rolled rod-AR 86%	1084 ± 7	1237 ± 19	20.0 ± 1.2
